# Tailoring Negative Thermal Expansion via Tunable Induced Strain in La–Fe–Si-Based Multifunctional Material

**DOI:** 10.1021/acsami.2c11586

**Published:** 2022-09-13

**Authors:** Rafael Oliveira Fleming, Sofia Gonçalves, Amin Davarpanah, Iliya Radulov, Lukas Pfeuffer, Benedikt Beckmann, Konstantin Skokov, Yang Ren, Tianyi Li, John Evans, João Amaral, Rafael Almeida, Armandina Lopes, Gonçalo Oliveira, João Pedro Araújo, Arlete Apolinário, João Horta Belo

**Affiliations:** † Institute of Physics of Advanced Materials, Nanotechnology and Nanophotonics (IFIMUP), Departamento de Física e Astronomia da Faculdade de Ciências da Universidade do Porto, Rua do Campo Alegre, 687, 4169-007 Porto, Portugal; ‡ Institute of Material Science, Technical University of Darmstadt, 64287 Darmstadt, Germany; § Department of Physics, City University of Hong Kong, Kowloon 999077 Hong Kong, China; ∥ X-ray Science Division, Argonne National Laboratory, Lemont, Illinois 60439, United States; ⊥ Department of Physics and CICECO, University of Aveiro, Universitary Campus of Santiago, 3810-193 Aveiro, Portugal; # Department of Chemistry, Durham University, South Road, Durham DH1 3LE, United Kingdom

**Keywords:** negative thermal expansion, strain, microstructuring, phase transitions, magnetocaloric materials, multifunctional materials

## Abstract

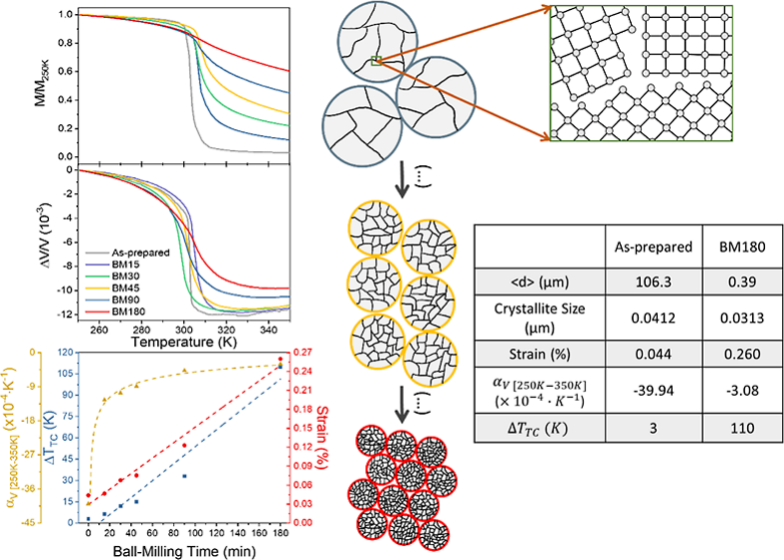

Zero thermal expansion (ZTE) composites are typically designed by combining positive thermal expansion (PTE) with negative thermal expansion (NTE) materials acting as compensators and have many diverse applications, including in high-precision instrumentation and biomedical devices. La(Fe_1–*x*_,Si_*x*_)13-based compounds display several remarkable properties, such as giant magnetocaloric effect and very large NTE at room temperature. Both are linked via strong magnetovolume coupling, which leads to sharp magnetic and volume changes occurring simultaneously across first-order phase transitions; the abrupt nature of these changes makes them unsuitable as thermal expansion compensators. To make these materials more useful practically, the mechanisms controlling the temperature over which this transition occurs and the magnitude of contraction need to be controlled. In this work, ball-milling was used to decrease particles and crystallite sizes and increase the strain in LaFe_11.9_Mn_0.27_Si_1.29_H_*x*_ alloys. Such size and strain tuning effectively broadened the temperature over which this transition occurs. The material’s NTE operational temperature window was expanded, and its peak was suppressed by up to 85%. This work demonstrates that induced strain is the key mechanism controlling these materials’ phase transitions. This allows the optimization of their thermal expansion toward room-temperature ZTE applications.

## 1 Introduction

Currently, there is an urge to mechanically control materials at the micro- and nanoscale in a wide set of technological industries, for example, micro-devices, large telescopes, dentistry, sensors, and fuel cells.^1−7^ Small mechanical distortions originating from materials’ thermal expansion can degrade high-precision devices and instruments, making the accurate control of materials’ and devices’ thermal expansion of fundamental interest. This issue is particularly problematic for devices with several constituents (such as multilayered devices) where there is likely to be a thermal expansion mismatch between their constituent materials.^3,4,7−9^ Such thermal expansion mismatch between materials can cause mechanical cracks and disrupted electrical connections. In such cases, controllable thermal expansion can enhance device reliability and prolong their lifetime.^4−6,8^ In order to tackle this technological problem, materials and/or composites with near-zero thermal expansion behavior [zero thermal expansion (ZTE) or invar materials] are required.^5,7,9^

While most materials expand when heated, exhibiting a positive thermal expansion (PTE), some possess an unusual property of volume contraction—these are said to have a negative thermal expansion (NTE).^1−5,8,10^ Functional composites’ thermal expansion can be tailored by combining PTE with NTE constituent materials. In particular, ZTE composites can be achieved by combining materials with isotropic thermal expansions, where the NTE material will act as a thermal expansion compensator.^5,7,11−13^ Therefore, it is extremely important to understand the underlying intrinsic chemical–physical behavior of these NTE materials and provide fundamental insights, so that they are controllable for a wide range of applications.^14−28^

Among the different mechanisms that lead to NTE behavior,^1−5,8,10^ one is the occurrence of a magnetovolume coupling.^17,29−32^ This mechanism typically arises from a simultaneous magnetic and atomic lattice transition, where the large magnetization variation at the Curie temperature (*T*_*C*_) is accompanied by a large change in the crystal structure. The simultaneous variation of two order parameters (magnetization and unit cell volume) typically occurs in materials with first-order phase transitions such as the LaFeSi, HfNbFe, and MnZnN material families.^4,5^ This coupling provides the ability to tune the thermal expansion of these materials by adjusting their magnetic properties.

More recently, another mechanism controlling the thermal expansion has been attracting research attention: the size confinement of any given material (typically when one of the spatial dimensions is smaller than 100 nm). Such microstructural downsizing can lead to the appearance of novel physical properties due to size-induced effects.^13,16^ In particular, several materials that present bulk PTE have been found to exhibit NTE when presenting at least one dimension confined at the nanoscale.^2,13,16,19,33^ These materials’ properties, such as magnetization, superficial/interfacial coordination, local lattice symmetry, and elemental distribution, can be changed through size reduction, enabling the possibility of tailoring associated features such as their thermal expansion.^13,16^

The La(Fe_1–*x*_,Si_*x*_)_13_-derived compounds are very well known for their giant magnetocaloric effect and NTE behavior.^9,10,14,15,17,18,34−46^ Their crystal structure is cubic with Fm-3c space group, and they display a ferromagnetic-to-paramagnetic (FM-PM) isostructural transition, corresponding to a low-temperature high-magnetization (ordered) state that evolves into a high-temperature low-magnetization (disordered) state as the temperature is increased across their *T*_C_.^26^ Along these transitions, the crystal structure is retained, but the unit cell volume contracts by 3000–30,000 ppm typically over a <50 K temperature interval.^5,9,15,35−37^ To act as compensators, NTE materials should have a more gradual change of volume over a larger temperature window, akin to the typical changes on standard PTE materials. Fortunately, it is possible to tune and shape the profile of the magnetization versus temperature curves (namely, the *T*_C_ and the *dM*/*dT*) by tuning both extrinsic (pressure and magnetic field)^35,42−46^ and intrinsic (stoichiometry) properties.^9,10,18,35,38−41^ Recently, there have been remarkable examples of thermal expansion control via chemical substitutions, where the typically abrupt NTE behavior is smoothed across a wider temperature window.^9,10,18,35,38−41^ For example, by replacing Fe by Co and Mn, or by adjusting the Si/Fe ratio, or even by electrolytic hydrogenating La–Fe–Si, Li’s group has successfully been able to tune the operational temperature and smooth out La–Fe–Si sharp volume transition.^9,15,24−26^

This work proposes an alternative mechanism to control the thermal expansion of strongly magnetovolume coupled materials—microstructural strain induced by ball-milling (BM).^47,48^ It is known that magnetic disorder (which can be induced by microstructural strain) has a strong impact on the magnetic transition profile: increasing magnetic disorder leads to a broadening of the magnetic transition that is associated with a wider temperature distribution of Tc’s.^47,48,56,59^ Hence, due to La–Fe–Si strong magnetovolume coupling, in this work, we propose to make use of the microstructural strain to control the magnetic transition profile and consequently tune its structural transition and thermal expansion coefficient. Moreover, the size reduction effects on LaFe_11.9_Mn_0.27_Si_1.29_H_*x*_ morphological, microstructural, magnetic, and thermal expansion properties were thoroughly characterized and correlated, providing a comprehensive physical model for the observed behaviors.

## 2 Experimental Information

**Figure 1:**
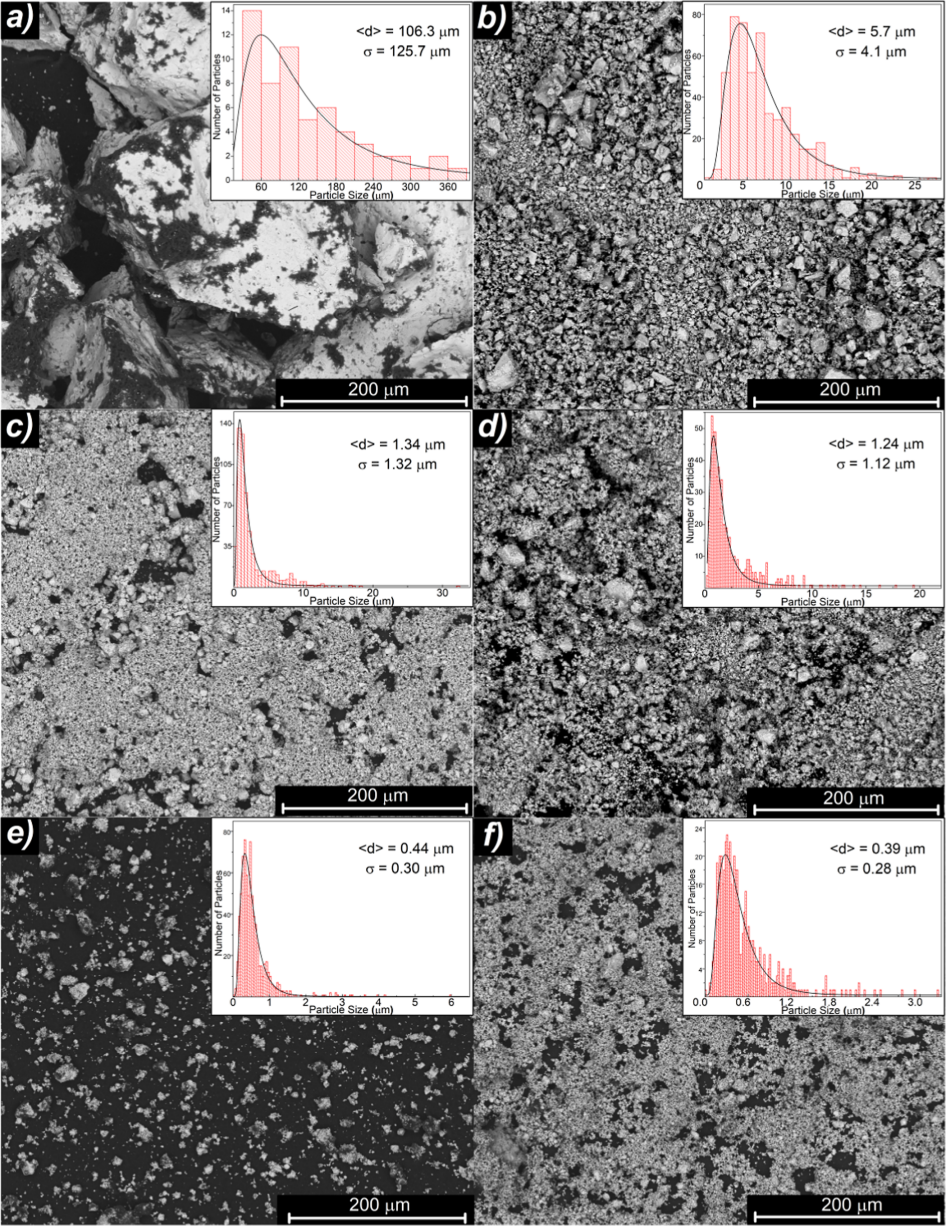
SEM images obtained with backscattered electron mode and size distribution histograms of LaFe_11.9_Mn_0.27_Si_1.29_H_*x*_ for the (**a)** as-prepared, (**b)** BM15, (**c)** BM30, (**d)** BM45, (**e)** BM90, and (**f)** BM180 LaFe11.9Mn0.27Si1.29Hx samples.

The LaFe_11.9_Mn_0.27_Si_1.29_H_*x*_ sample was prepared by arc-melting followed by hydrogenation.^49^ After melting, to ensure homogeneity of the as-cast alloy, the ingot was encapsulated in quartz tubes under an Ar atmosphere and then annealed at 1050 °C for 7 days and subsequently quenched in water. Hydrogenation was carried out on 1–2 mm fragments of the parent bulk sample in a furnace filled with 0.9 bar H_2_ atmosphere, at 723 K for 1 hour, to saturate the H concentration. The as-prepared hydrogenated pieces of LaFe_11.9_Mn_0.27_Si_1.29_H_*x*_ alloy were hand-milled for a short period of time, resulting in an initial set of particles with a 100 μm average size and a size distribution width (FWHM) of 126 μm (Figure 1a). This sample is termed “as-prepared” throughout this article. Roughly 2 g of the as-prepared sample was placed inside a zirconia vessel together with four zirconia spheres of 5 mm radius and 38 g mass. No liquid media were included in the vessel. The ball-to-powder mass ratio was estimated to be 76. The vessel was installed in a FRITSCH PULVERISETTE 23 Mini Mill, and the powder was milled with a vertically oscillating frequency of 50 Hz. Every 5 min, the milling was paused for 5 min to prevent any overheating of the vessel and the powder. The total milling time was 180 min, and at 15, 30, 45, 90, and 180 min, roughly 100 mg of the LaFe_11.9_Mn_0.27_Si_1.29_H_*x*_ milled powder was extracted and thoroughly characterized. These different powder samples have been termed, respectively, as BM15, BM30, BM45, BM90, and BM180. Every sample’s chemical, micro, atomic structure, magnetic, and thermal expansion properties were characterized as described below. All samples’ morphology and chemical composition were investigated by scanning electron microscopy (SEM) and energy dispersive spectroscopy (EDS) using FEI Quanta 400FEG ESEM/EDAX Genesis X4M equipment. SEM image statistical analysis was performed using the ImageJ open source software.^50^ Several hundreds of particles from the SEM images were randomly chosen for each sample to determine the particle size distribution, namely, the average particle size and the size standard deviation. Magnetic characterizations of the powder samples were performed in a commercial Superconducting Quantum Interference Device (SQUID) magnetometer (MPMS3, by Quantum Design) by measuring their magnetization as a function of temperature between 350 and 5 K while cooling with a magnetic field of 1000 Oe and by measuring isothermally their magnetization as a function of magnetic field at 20 K. All magnetic measurements were taken under identical geometries and were properly corrected for inevitable deviations to the dipole approximation in accordance with the recent report by Amorim and co-authors.^62^

The room-temperature crystal lattice parameters were obtained by Rietveld refinements against X-ray diffractograms measured in a Rigaku SmartLab using a Cu X-ray source (Kα1 = 1.5406 Å). The samples’ thermal expansion was evaluated through Rietveld refinements of X-ray diffractograms obtained while varying the temperature between 250 and 350 K at 1 K/min under heating and cooling, with each diffractogram corresponding to a 1 K step. These measurements were performed with synchrotron radiation (λ = 0.1173 Å) at the Advanced Photon Source (APS) of the Argonne National Laboratory (beamline 11-ID-C). Each powder sample was wrapped in a Cu thin foil such that the diffraction pattern includes reflections linked to the cubic Cu phase. As Cu thermal expansion is very well known,^51^ a conversion of any given unit cell volume to temperature can be made, which results in Cu working as a local sample thermometer.

## 3 Results and Discussion

### 3.1 Morphology

Figure 1 shows the SEM images of all the samples and their corresponding particle size distributions and lognormal fits. We have obtained a 96% average particle size reduction from 106 μm for the as-prepared sample to 0.39 μm for the maximum BM time, *t*_BM_, of 180 min, together with a 98% reduction of the standard deviation.

### 3.2 Phase Composition and Crystal Structure

LaFe_11.9_Mn_0.27_Si_1.29_H_*x*_ has the NaZn13-type crystal structure (space group Fm-3c), where the Fe atoms, 8b (Fe(I)) and 96i (Fe(II)), occupy two nonequivalent 8b (Fe(I)) and 96i (Fe(II)) sites; Si atoms preferentially occupy Fe(II) sites;^11,35^ Mn atoms tend to occupy equally both Fe(I) and Fe(II) sites;^38,39^ and the hydrogen atoms lie at interstitial sites^41^ (see Figure S1 in the Supporting Information). The Rietveld refinements against all X-ray diffractograms at 273.6 K reveal the expected NaZn_13_-type crystal structure and a minority phase fraction of α-Fe (space group *Im-3m*), which is commonly observed in samples prepared by this route.^40^ An X-ray diffractogram with a Rietveld refinement of sample BM15 is presented in Figure S2 (Supporting Information). In addition, peaks due to the Cu cubic phase (space group *Fm-3m*) used for sample wrapping are identified and fitted.

**Figure 2:**
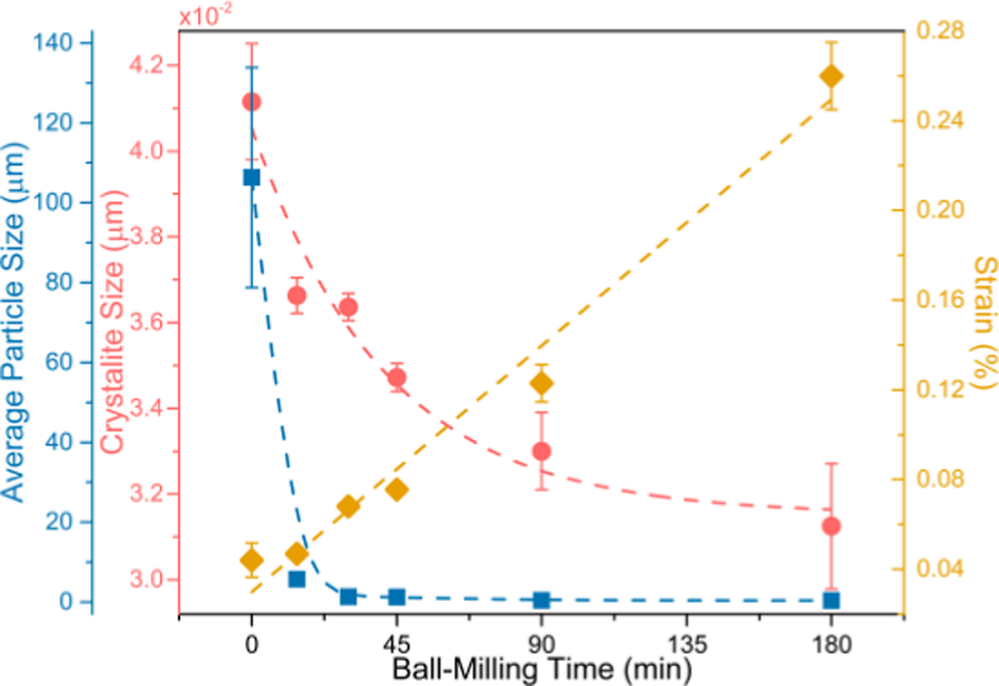
Average particle size (blue squares), crystallite size (red circles), and strain (yellow rhombus) of LaFe_11.9_Mn_0.27_Si_1.29_H_*x*_ samples as a function of ball-milling time. The error bars of the average particle size that are not visible are contained within the data square area.

Figure 2 displays the average particle size, crystallite size, and strain as a function of *t*_BM_. The average particle size was obtained by SEM statistical image analysis. In contrast, the crystallite size (*D*) and strain (ε) were obtained through a linear regression following the Williamson–Hall relationship [see Figure S3 (Supporting Information)]
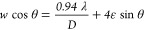
1where *w* is the FWHM of the XRD peaks corresponding to the La(Fe_1–*x*_,Si_*x*_)_13_ structure_,_ λ is the incident X-ray wavelength, and θ is the peak position.^52−55^

Increasing *t*_BM_ leads to a decrease in average particle and crystallite sizes following a decreasing trend (Figure 2). Comparing the as-prepared and BM180 samples, the crystallite size decreases from 41.1 to 33.0 nm. Simultaneously, BM induces strain, which can be caused by the higher number of crystallites (and correspondingly a higher number of grain boundaries), high induced chemical disorder, grain surface relaxation, and nonuniform lattice distortions.^42,43,46,55^ Additionally, one can observe that both particle size and crystallite size display an inverse trend in comparison with the strain.^55^ Strain increases linearly with *t*_BM_, from 0.044% (for the as-prepared sample) to 0.26% (for the BM180 sample). A linear fit to this behavior yields a 1.22 × 10^–5^ slope, meaning that a strain of ∼1.22 × 10^–3^% is induced per every minute of BM. Thus, by relative comparison, it is possible to conclude that BM time has a greater impact on the strain than on the crystallite size.

### 3.3 Magnetic Transition

**Figure 3:**
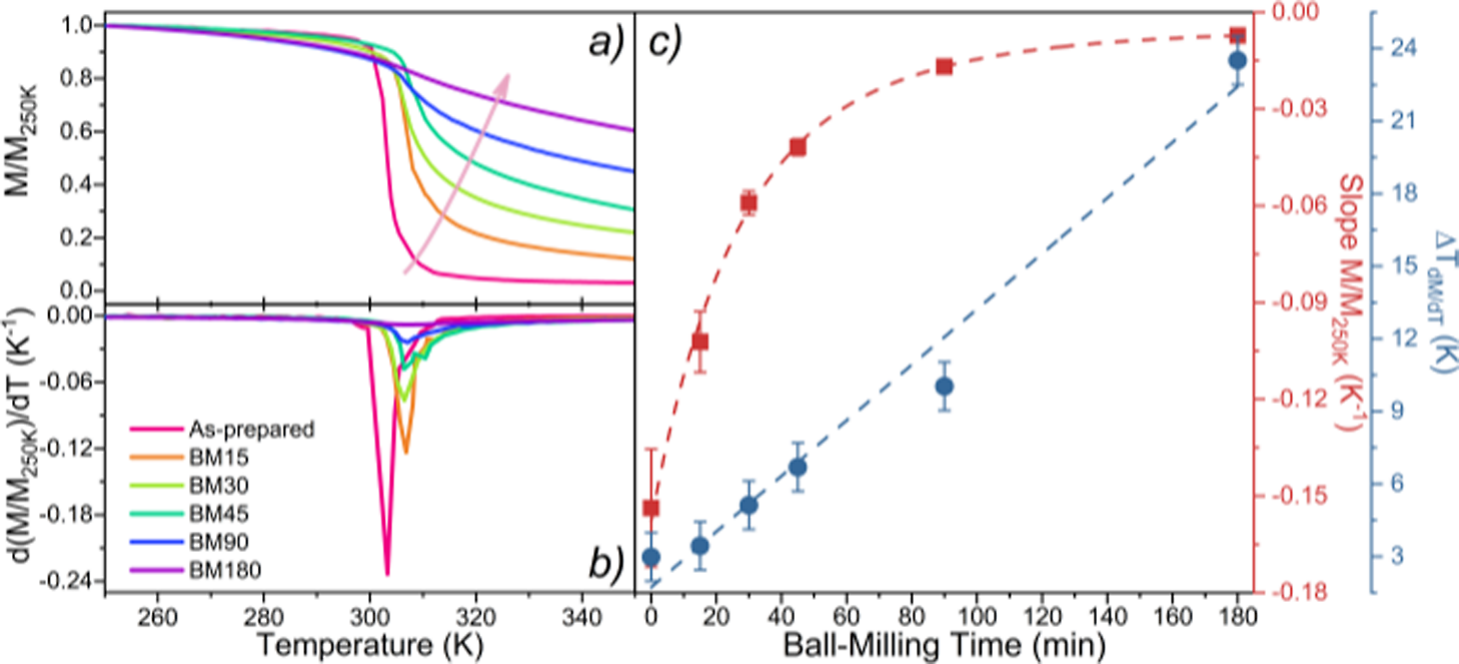
(**a)** Normalized magnetization, (M(T)/M(T = 250 K)), M/M_250K_, and (**b)** d*M*/d*T*(*T*) of the as-prepared, BM15, BM30, BM45, BM90, and BM180 LaFe_11.9_Mn_0.27_Si_1.29_H_*x*_ samples, measured in cooling with an applied field of 1 KOe. (**c)** Slope of the *M*(*T*) curves within the FWHM of the d*M*/d*T* curve (red squares) and the corresponding temperature window width of the FWHM, Δ*T*_d*M*/d*T*_ (blue dots), as a function of ball-milling time.

Because the NTE behavior of La(Fe_1–*x*_,Si_*x*_)_13_-based compound family is strongly correlated with its magnetic transition, magnetization vs temperature measurements were performed in all samples. Figure 3a shows the temperature dependence of the normalized magnetization (M(T)/*M*(*T* = 250 K)), M/M_250K_ measured on cooling and with an applied magnetic field of 1 kOe, in the temperature range from 250 to 350 K for all samples. Figure 3b shows the derivative of the *M*(*T*) curves, d*M*/d*T*(*T*). From these curves, one can extract the full width at half maximum (FWHM = Δ*T*_d*M*/d*T*_) and the absolute value of their global minimum peaks, d*M*/d*T*_peak_. Figure 3(c) shows the Δ*T*_d*M*/d*T*_ as a function of *t*_BM_ and also the slope of the *M*(*T*) curves, M/MT = 250 K, evaluated in the respective temperature range corresponding to Δ*T*_d*M*/d*T*_.

The magnetization increases on cooling, revealing the typical PM–FM phase transition at *T*_C_. The as-prepared sample presents a sharp transition at *T*_C_ ∼ 305 K, and its magnetization then remains nearly constant with further temperature decrease. In comparison, the *M*(*T*) profiles of the BM samples present an increasingly smoother transition with increasing *t*_BM_. In fact, the M(T_350K_)/M(*T* = 250 K) value increases monotonously as the *t*_BM_ increases, indicating that the FM–PM transition becomes more gradual with *t*_BM_.

The d*M*/d*T*_peak_ quantifies the “abruptness” of the phase transition (also known as the thermomagnetic coefficient) and indicates the temperature at which the magnetization is changing most drastically, corresponding to *T* ∼ *T*_C_. Its dependence on *t*_BM_ is represented in Figure S4 (Supporting Information) where a reduction from ∼0.23 *K*^–1^ (as-prepared) to ∼0.01 *K*^–1^ (BM180) is observed.

The Δ*T*_d*M*/d*T*_ quantifies how broad the magnetic transitions are across temperatures. This quantity varies approximately linearly with *t*_BM_—increasing from ∼3 K (as-prepared) to ∼24 K (BM180), which shows how the broadening of the magnetic transition evolves with *t*_BM_.

The slope of the *M*(*T*) curves decreases rapidly with *t*_BM_—decreasing from ∼0.15 K^–1^ (as-prepared) to ∼0.01 K^–1^ (BM180). This means that the temperature-induced magnetization variation is strongly suppressed as *t*_BM_ increases. In addition, the profile of the slope of the *M*(*T*) curves vs *t*_BM_ (Figure 3(c)) shows an exponential-like behavior, as its values decay until a plateau region is reached—suggesting that increasing *t*_BM_ further will not promote significant slope changes.

#### 3.3.1 Bean–Rodbell Analysis

From a theoretical point of view, the Bean–Rodbell model has been particularly successful in explaining and predicting the magnetic transitions of materials with strong magnetovolume coupling.^56−59^ This model relies on the simple and observable assumption that the *T*_C_ of a strongly coupled magnetovolume material depends on the unit cell volume (hence strain) via a β coefficient (proportional to d*T*_*C*_/d*V*). In addition, this model also introduced a new term η, which is dependent on β and on the material compressibility (*K*), allowing us to quantitatively distinguish materials with strong (η > 1) and weak (η < 1) magnetovolume coupling. The *M*(*T*) curves were fitted (see Figure S5 in the Supporting Information) using the Bean–Rodbell model, assuming that the transition can be described not by a single *T*_C_, but instead by a lognormal distribution of *T*_C_’s (associated with the induced microstrain), with a given width, , assuming fixed values for spin (0.92) and η (1.8)—in accordance with refs (63)(64),. As can be seen in Figure S6 in the Supporting Information,  increases drastically with *t*_BM_ from ∼3 K (as-prepared) up to ∼110 K (BM180). Similar to the strain and Δ*T*_d*M*/d*T*_,  displays a linear relationship with *t*_BM_.

### 3.4 Thermal Expansion Characterization

**Figure 4:**
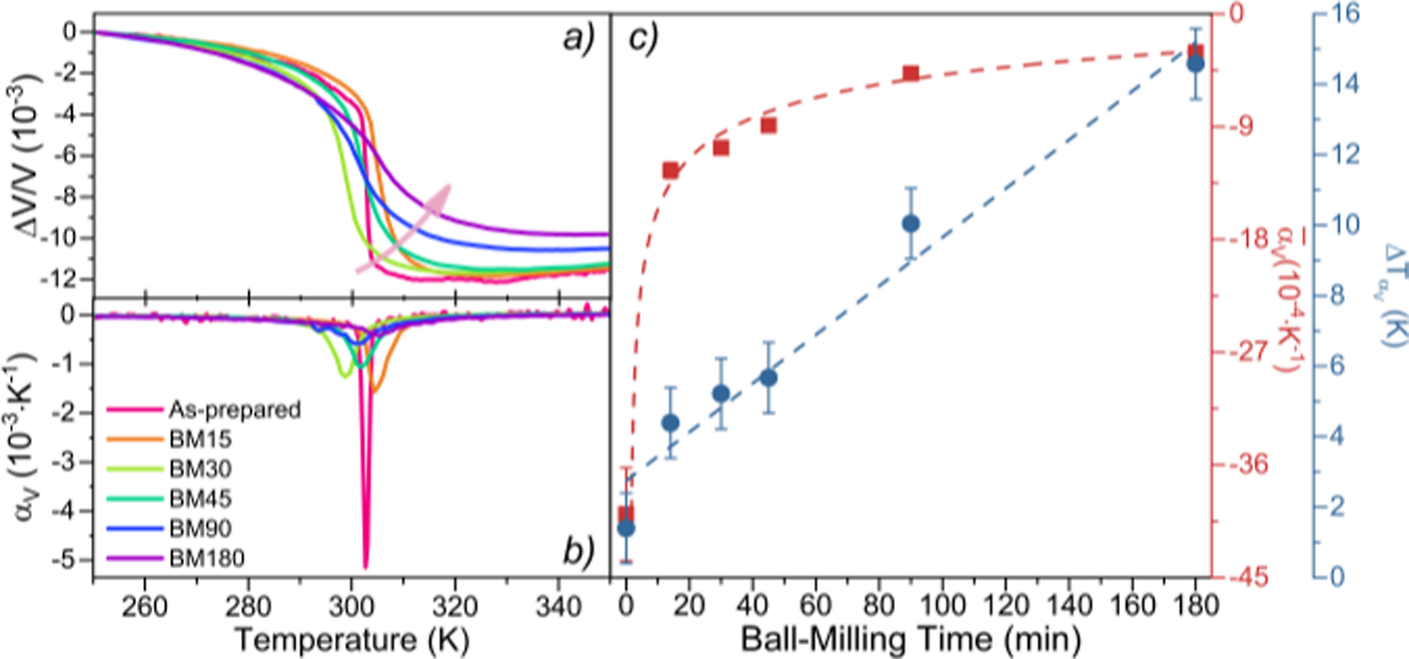
(**a)** Δ*V*/*V*(*T*) and (**b)** α_*V*_ of the as-prepared, BM15, BM30, BM45, BM90, and BM180 LaFe_11.9_Mn_0.27_Si_1.29_H_*x*_ samples, measured in cooling as a function of temperature. (**c)** (red dots), evaluated in , and the value of the  (blue dots) as a function of the ball-milling time.

Figure 4a shows the relative variation of the volume of the unit cell as a function of the temperature Δ*V*(*T*)/*V*(*T* = 250 K)= [*V*(*T*) − *V*(*T* = 250 K)]/*V*(*T* = 250 K). Figure 4b shows the temperature derivative of the Δ*V*(*T*)/*V*(*T* = 250K) curves, which is the equivalent to the coefficient of thermal expansion as a function of temperature, α_*V*_(*T*). From this curve, one obtains the FWHM, , and the value of its global minimum peak, α_V_(*T*)_peak_. Figure 4c shows the value of the linear thermal expansion coefficient,  evaluated in a temperature window defined by  and  as a function of *t*_BM_.

Δ*V*(*T*)/*V*(*T* = 250K) curves show that the volume decreases with the increasing temperature, that is, α_*V*_ < 0, demonstrating NTE behavior across the transition. In addition, the evolution of the Δ*V*(*T*)/*V*(*T* = 250K) curves’ profiles with *t*_BM_ is very similar to the evolution of the *M*(*T*) curves’ profiles: a gradual broadening of the transitions, as a result of the strong magnetovolume coupling. In addition, the overall Δ*V*/*V* variation tends to diminish with *t*_BM_: the as-prepared sample has an overall Δ*V*/*V* of ∼11.5 × 10^–3^, whereas the BM180 has ∼9.8 × 10^–3^.

The α_*V*_(*T*)_peak_ dependency with *t*_BM_ is shown in Figure S7 (Supporting Information), where a value of ∼2210 × 10^–6^ K^–1^ is observed for the as-prepared sample and a value of ∼260 × 10^–6^ K^–1^ is obtained for the BM180 sample.

 shows a linear relation, with *t*_BM_ increasing from ∼1 K (as-prepared) to ∼15 K (BM180). Similar to what was observed with the magnetic transition, the  decreases rapidly with *t*_BM_ from ∼40 × 10^–4^ K^–1^ (as-prepared) to ∼3 × 10^–4^ K^–1^ (BM180). Additionally, the  tends to decrease rapidly with *t*_BM_, reaching then a plateau region. As with the magnetization slope, this behavior suggests that further BM will not induce significant  value changes in the temperature range defined. Hence, the  and  behaviors clearly demonstrate that *t*_BM_ is linearly expanding the temperature interval where the transition occurs and, concomitantly, making it less sharp.

The α_*V*_(*T*) was also analyzed in the whole temperature interval, from 250 to 350 K, . Figure S8 (Supporting Information) shows the relation between  and *t*_BM_, where a linear relation is observed. The as-prepared sample shows a  of ∼ −115 × 10^–6^ K^–1^ and the BM180 a  of ∼ −98 × 10^–6^ K^–1^.

**Figure 5:**
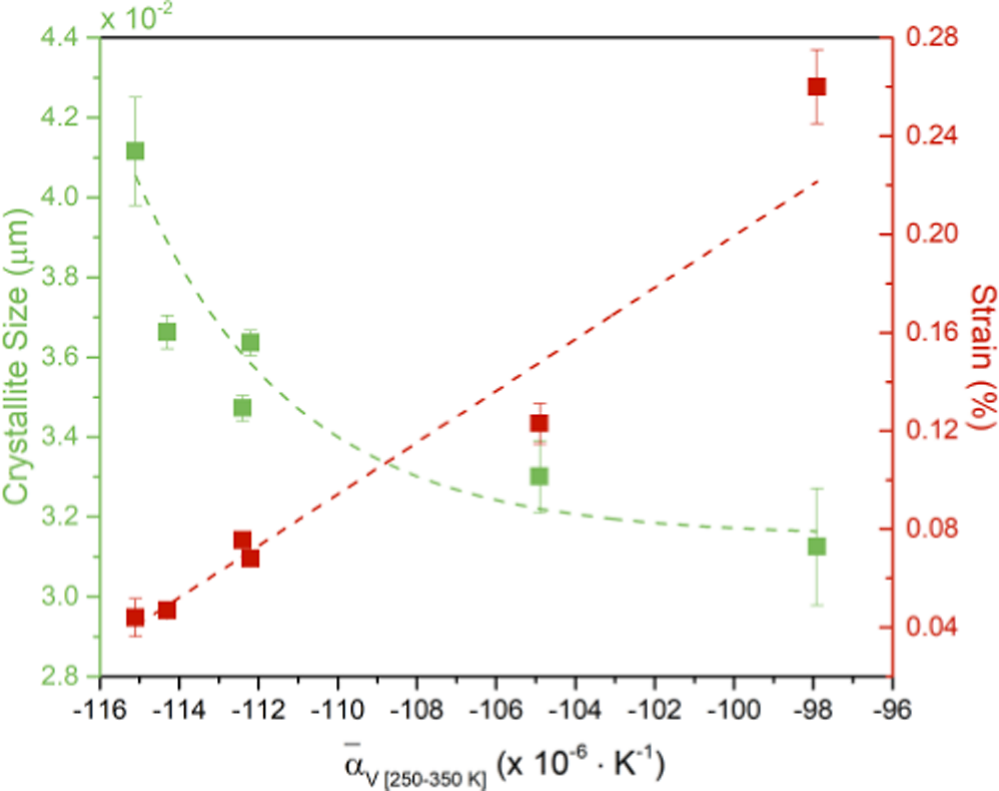
Crystallite size (green squares) and strain (red circles) dependence with the  of all the samples.

Figure 5 shows the relation between the crystallite size and strain with the linear . Because  is proportional to *t*_BM_, the relation between  and the crystallite size and strain is similar to the relation between these two variables and *t*_BM_, as shown in Figure 2.

In order to try to disentangle the roles of crystallite size and induced microstrain on the observed changes of the magnetic and structural transitions with milling time, the raw patterns, the unit cell volume, the Fe^I^–Fe^II^ interatomic distances (known as the interatomic distance influencing *T*_c_ the most),^15,37,60^ and the *T*_c_ were plotted against ball-milling time as can be seen in Figures S13–15 in the Supporting Information. The absence of significant or monotonous changes observed in the center peak position, unit cell volume, Fe^I^–Fe^II^ interatomic distances, or *T*_c_ as a function of milling time, coupled with the clear, symmetric, and linear broadening of the diffraction and magnetization derivative peaks, strongly suggests that the linearly increasing microstrain has the main role on tuning the magnetic and structural transitions. This physical picture is further corroborated by the Bean–Rodbell analysis, where a linearly increasing broadening of the *T*_c_ distribution is required to fit the measured *M*(*T*) curves as a function of milling time (Figure S6).

Additionally, in order to further untangle the effect of size confinement from that of induced microstrain, we have measured low-temperature (20 K), isothermal magnetization versus magnetic field curves for the as-prepared and BM180 samples, as plotted in Figure S12. As can be seen, the saturation magnetization remains nearly unchanged after the total 180 min of milling. This behavior contrasts with the behavior found for La–Fe–Si nanoparticles, where a 30% decrease in the saturation magnetization was observed.^61^ In fact, the suppression of the magnetization values, together with shift of the transition temperatures, is typically observed in a wide range of nanostructured magnetocaloric materials.^47^ Hence, these results demonstrate that the milling is not changing the individual magnetic moments but is instead inducing magnetic disorder (due to the structural disorder—microstrain) that manifests itself in broadened magnetic and structural phase transitions.

Interestingly, comparing the magnetic and structural transitions as seen in Figure S10, we can see that the relative variation in magnetization between 250 and 350 K is more pronounced than the relative change in unit cell volume. This suggests that the magnetic correlations are more sensitive to strain than the unit cell volume of the sample.

**Figure 6:**
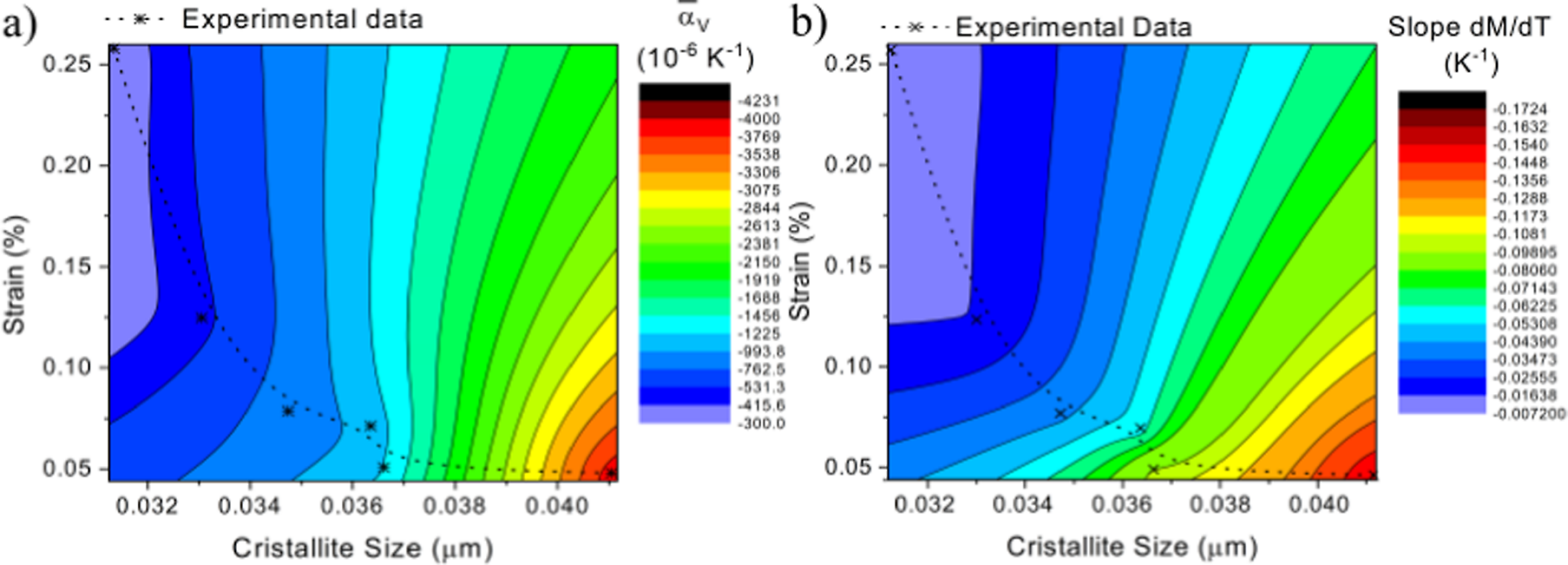
(a) Contour plot of the  color code (where red and blue represent larger and smaller absolute values, respectively) evaluated at the  temperature window as a function of strain and crystallite size. (b) Contour plot of the *M*(*T*) slope color code (where red and blue represent larger and smaller absolute values, respectively) evaluated at the Δ*T*_d*M*/d*T*_ temperature window as a function of strain and crystallite size. Both contour plots were performed by interpolation of the experimental values.

Figure 6a,b displays the contour plots (color maps) of the strain and crystallite size as a function of the slope parameters  and M/MT = 250K slope, respectively. We used (crystallite size, strain, ) and (crystallite size, strain, M(T)/M(T = 250 K) slope) experimental data for each sample (both of which are specified in Table 1) to perform a numerical estimation of 100 new data points by a method for interpolating spatial data. Gridding methods to convert XYZ data into a matrix were performed using the Kriging method algorithm.

**Table 1:** Most Relevant Values Used Throughout the Work: Ball-Milling Time (*t*_BM_ (min)); Strain (%); Crystallite Size (μm); Linear Coefficient of Thermal Expansion [( (×10––-6 K-–1)) )] Evaluated in the Temperature Range of 250 to 350 K; Curie Temperature (*T*_c_) (K); Full Width at Half Maximum (FWHM) of the *T*_c_ Distribution [ (K)); )]; FWHM of the αV curve ( (K)); FWHM of the dM/dT Curve (ΔTdM_/*dT*_ (K)); Linear Coefficient of Thermal Expansion Evaluated in the  ( (×10^–6^ K^–1^)]; Slope of the *M*(*T*) Curve Evaluated at the Δ*T*_d*M*/d*T*_ [M/MT = 250 K Slope (K^–1^)]; Average Particle Size (μm)

ball-milling time (min)	strain (%)	crystallite size (μm)	(×10^6^ K^–1^)	*T*C (K)	(K)	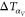 (K)	Δ*T*_d*M*/d*T*_ (K)	(×10^–6^ K^–1^)	*M*/*M*_*T* = 250__*K*_ slope (K^–1^)	average particle size (μm)
0	0.044	0.0412	–115.1	304	3	1.40	2.98	-3994.18	–0.154	106.30
15	0.047	0.0366	–114.3	307	6.5	4.39	3.44	-1251.41	–0.102	5.74
30	0.068	0.0364	–112.2	307	12	5.22	5.12	-1069.88	–0.059	1.35
45	0.076	0.0347	–112.4	306	15	5.67	6.69	–890.71	–0.042	1.24
90	0.123	0.0330	–104.9	307	33	10.05	10.04	–476.45	–0.017	0.44
180	0.260	0.0313	-97.9	307	110	14.58	23.51	–307.74	–0.007	0.39

Using this interpolation method, we are able to create an estimation for new values of  and M(T)/M(T = 250 K) slope for a range of strain and crystallite size values. The obtained (strain, crystallite size, ) and (strain, crystallite size, M(T)/M(T = 250 K) slope) matrices were then plotted in 3D contour plots represented in Figure 6a,b. One can observe that higher values of  coincide with higher values of crystallite size and lower values of strain, corresponding to samples that were not milled. As one decreases the crystallite size and continuously increases the strain, the  decreases drastically (nearly by 2 orders of magnitude). This further highlights the role of the strain and crystallite size on ruling the α_*V*_(*T*) of these materials while providing pathways for further thermal expansion coefficient tailoring. A remarkably similar color pattern is observed in Figure 6b, which underlines the resemblance of the magnetic and structural transitions and how they evolve similarly with controllable strain and crystallite sizes.

Table 1 summarizes the most relevant parameters evaluated throughout this work.

## 4 Conclusion

In summary, BM leads to a significant reduction of the average particle and crystallite size and to a drastic linear enhancement of the induced microstrain in LaFe_11.9_Mn_0.27_Si_1.29_H_*x*_. A severe broadening of the magnetization versus temperature profiles across *T*_C_ is observed, quantified by a 93% suppression of its M(T)/M(T = 250 K) slope and an 88% enhancement of the transition width (Δ*T*_d*M*/d*T*_). In the framework of the Bean–Rodbell model, such transition widening can be explained by the presence of an ever-wider *T*_C_ distribution as a consequence of the increased induced microstrain (via *t*_BM_). Due to strong magnetovolume coupling, both unit cell volume and magnetization dependence on temperature show a remarkably similar evolution with BM time. In particular, the α_*V*_(*T*)_peak_ decreases from ∼2210 (as-prepared) down to ∼ 260 ppm K^–1^ (after 180 min of BM), and the averaged α_*V*_ in the temperature range from 250 to 350 K, , decreases from −115 × 10^–6^ K^–1^ (as-prepared) to −98 × 10^–6^ K^–1^ (BM180).

This work demonstrates that the crystallite size and the ball-milled-induced microstrain (in particular the latter) are remarkably efficient tools to tune and customize the thermal expansion coefficient of La(Fe_1–*x*_,Si_*x*_)_13_-based compounds. Combining the method presented here with the usual chemical control method, a synergistic effect is expected toward the development of a near-zero thermal expansion material. In addition, this La(Fe_1–*x*_,Si_*x*_)_13_ size-control study triggers an opening research window for other NTE materials with strong magnetovolume coupling.

Finally, by inducing strain through a process like ball-milling, one is actively reducing the materials’ particle size, which demonstrates favorability for technological applications, because for the same filler content (%), smaller NTE particles are more effective in tuning (decreasing) a composite thermal expansion than the larger ones,^65−68^ enabling a controllable and homogenous compensating mechanism.

## Supporting Information Available

Schematic picture of LaFe_11.9_Mn_0.27_Si_1.29_H_*x*_ crystal structure; X-ray diffractogram of LaFe_11.9_Mn_0.27_Si_1.29_H_*x*_ at 273 K and the corresponding Rietveld refinement; Williamson–Hall analysis plot for the estimation of crystallite size (*D*) and strain (ε); evolution of the magnetization temperature derivative peak value as function of ball-milling time; as-prepared sample normalized magnetization versus temperature curve and the respective fit within the Bean–Rodbell model with a *T*_*c*_ distribution; evolution of *T*_*c*_ FWHM distribution and strain (%) with ball-milling time; peak value of thermal expansion coefficient as a function of ball-milling time; averaged (in the 250–350 K) thermal expansion coefficient as a function of ball-milling time; unit cell volume change and respective temperature derivatives and normalized magnetization and respective temperature derivatives for as-prepared and ball-milled samples for 45 and 180 min; relative change of unit cell volume and magnetization between 250 and 350 K; unit cell volume change and respective temperature derivative and normalized magnetization and respective temperature derivative for as-prepared sample plotted together with linear fittings within the FWHM temperature range; magnetization versus magnetic field curves measured at 20 K of the as-prepared and ball-milled samples for 180 min; raw XRD patterns for the as-prepared and ball-milled samples for 180 min; evolution of the unit cell volume and Fe^I^–Fe^II^ interatomic distances at 250 and 350 K as a function of ball-milling time; and averaged Curie temperature and FWHM of the magnetization derivative peak as a function of ball-milling time (PDF)
