# Supplementary material for: Tailoring Negative Thermal Expansion via Tunable Induced Strain in La–Fe–Si-Based Multifunctional Material
Source: ACS Appl Mater Interfaces. 2022 Sep 13;14(38):43498–507. doi: 10.1021/acsami.2c11586 (PMC9773235; doi:10.1021/acsami.2c11586)
Supplement: Supplementary file 1 — am2c11586_si_001.pdf [file am2c11586_si_001.pdf]

SUPPLEMENTARY INFORMATION

Tailoring negative thermal expansion via tunable induced strain in La-Fe-Si-based multifunctional material

Rafael Oliveira Fleming, Sofia Gonçalves*, Amin Davarpanah, Iliya Radulov, Lukas Pfeuffer, Benedikt Beckmann, Konstantin Skokov, Yang Ren, Tianyi Li, John Evans, Joao Amaral, Rafael Almeida, Armandina Lopes, Gonçalo Oliveira, João. Pedro Araújo, Arlete Apolinário, Joao Horta Belo⧫*

**both authors contributed equally to this work*

⧫ corresponding author e-mail: jbelo@fc.up.pt

R. O. Fleming*, S. Gonçalves*, A. Lopes, G. Oliveira, R. Almeida, J. P. Araújo, A. Apolinário and J. H. Belo⧫

Institute of Physics of Advanced Materials, Nanotechnology and Nanophotonics (IFIMUP), Departamento de Física e Astronomia da Faculdade de Ciências da Universidade do Porto, Rua do Campo Alegre, 687, 4169-007 Porto, Portugal

A. Davarpanah, I. Radulov, L. Pfeuffer, B. Beckmann and K. Skokov

Institute of Material Science, Technical University of Darmstadt, 64287 Darmstadt, Germany

A. Davarpanah, J. Amaral

Department of Physics and CICECO, University of Aveiro, Universitary Campus of Santiago, 3810-193 Aveiro, Portugal

Y. Ren

Department of Physics, City University of Hong Kong, Kowloon, Hong Kong, China

T. Y. Li

X-ray Science Division, Argonne National Laboratory, Lemont, IL 60439, USA

J. S. O. Evans

Department of Chemistry, Durham University, South Road, Durham DH1 3LE, United Kingdom

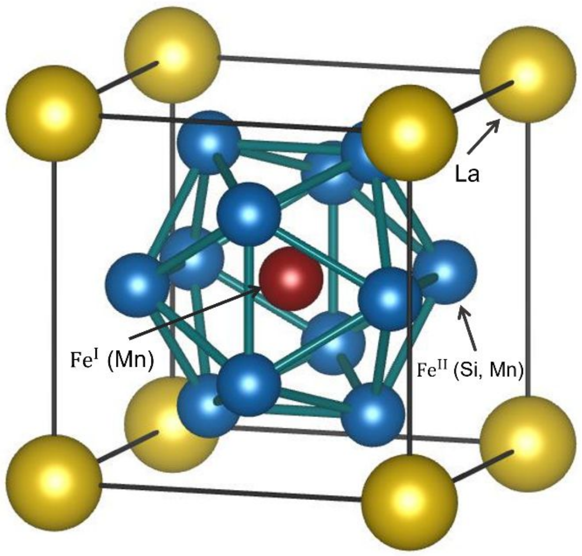

Figure S1. NaZn13-type crystal structure (space group Fm-3c) of $LaFe_{11.9}Mn_{0.27}Si_{1.29}H_x$ compounds. Mn atoms tend to occupy both Fe(I) and Fe(II) sites and hydrogen atoms are located in interstitial sites within the unit cell.

\

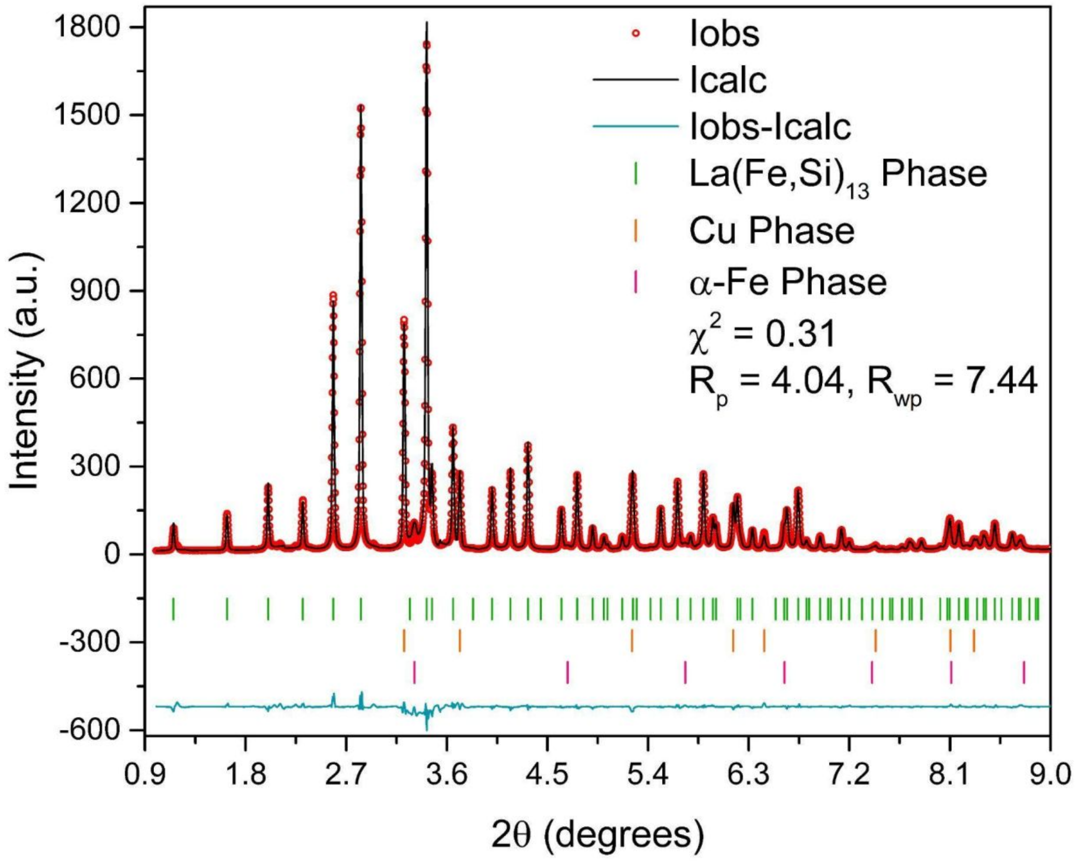

Figure S2. X-Ray diffractogram of sample BM14 performed at 273.55 K where a second phase of Fe is visible due to a small amount of α-Fe present in the sample and a cubic Cu phase is also visible due to the sample holder. All the diffractograms were measured using synchrotron radiation (λ = 0.1173 Å).

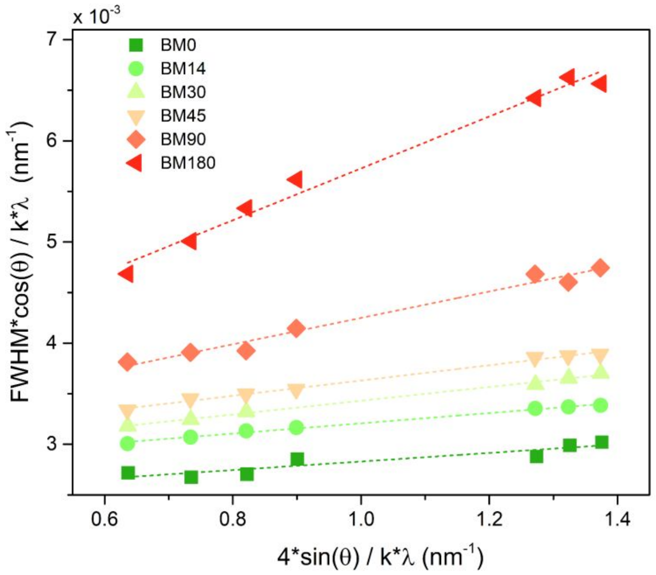

Figure S3. Williamson-Hall analysis of $LaFe_{11.9}Mn_{0.27}Si_{1.29}H_x$ diffracted peaks from all the samples diffractograms measured at 333 K.

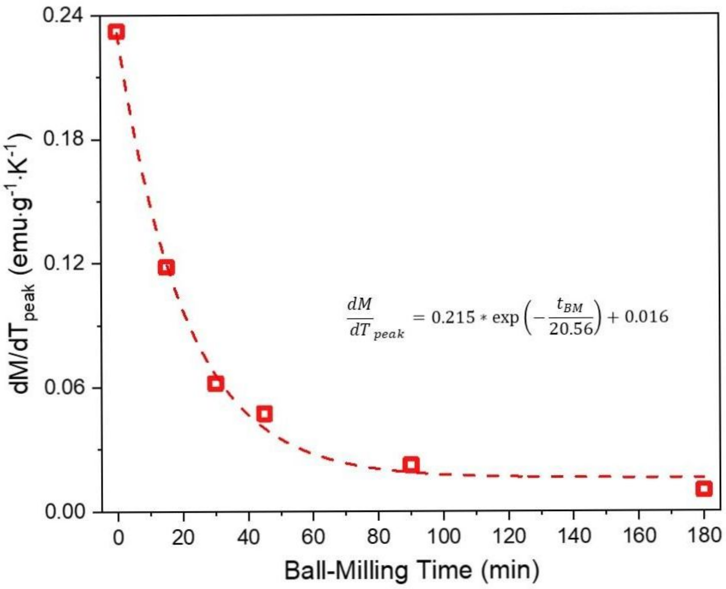

Figure S4. dM/dT $(T = 250\ K)$ peak height of $LaFe_{11.9}Mn_{0.27}Si_{1.29}H_x$ samples as a function of ball-milling time.

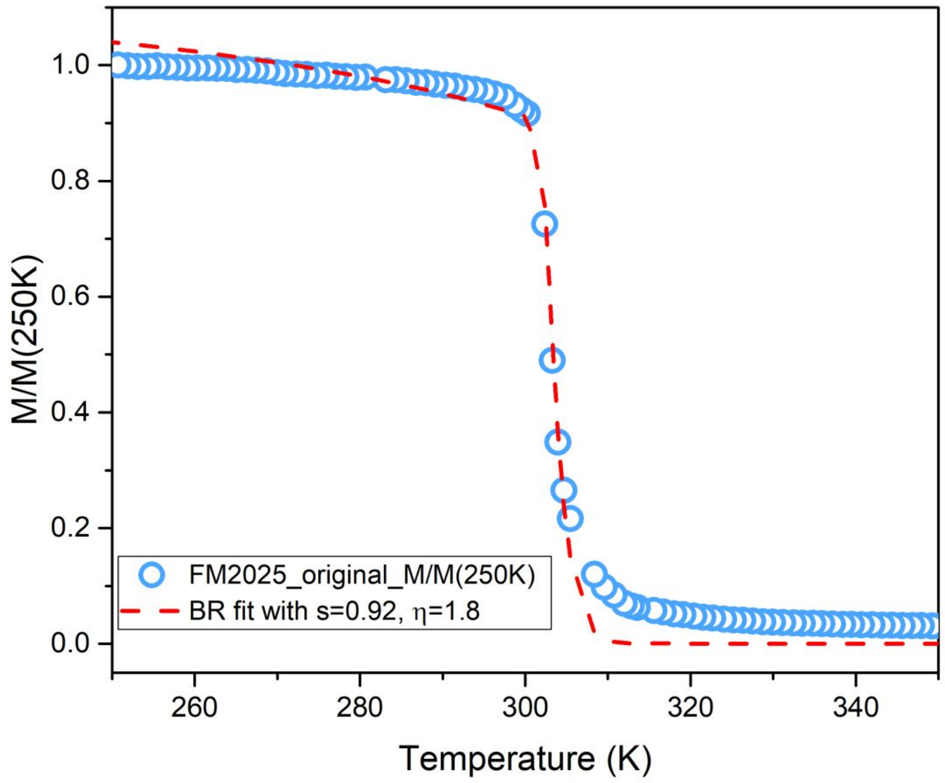

Figure S5. Bean-Rodbell fit to the $M/M_{T=250K}$ of the as-prepared sample of $LaFe_{11.9}Mn_{0.27}Si_{1.29}H_x$, assumed fixed values for spin (0.92) and η (1.8).

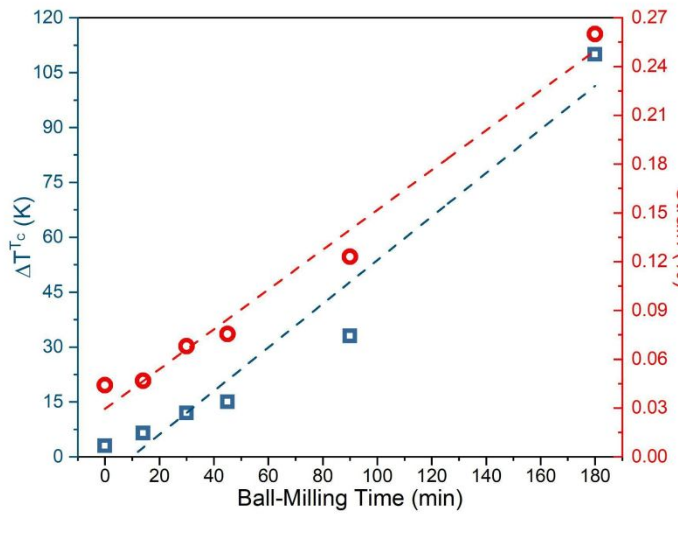

Figura S6. ΔT^{T_c} ($slope = 0.59558 \pm 0.06882$) (blue squares) and strain (%) ($slope = 0.00122 \pm 9.767 \times 10^{-5}$) (red circles) as a function of ball-milling time.

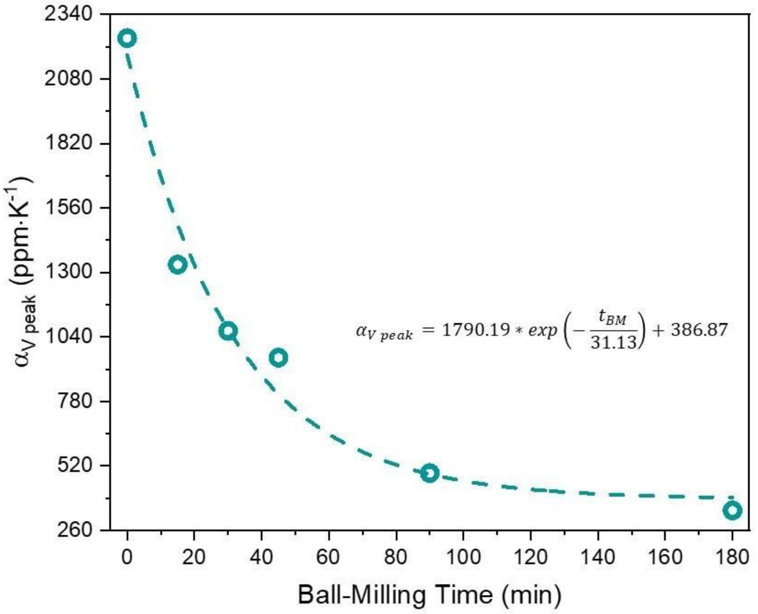

Figure S7. $\alpha_V(T)_{peak}$ as a function of ball-milling time with corresponding exponential fit.

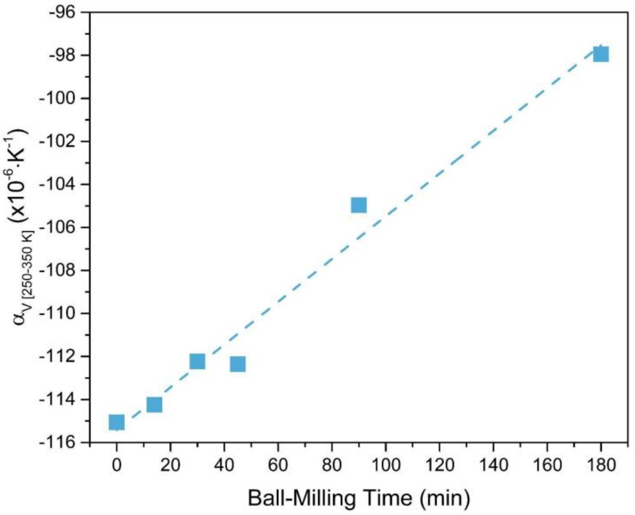

Figure S8. $\underline{\alpha}_{V\ [250-350\ K]}$ in the temperature range from 250 to 350 K as a function of ball-milling time.

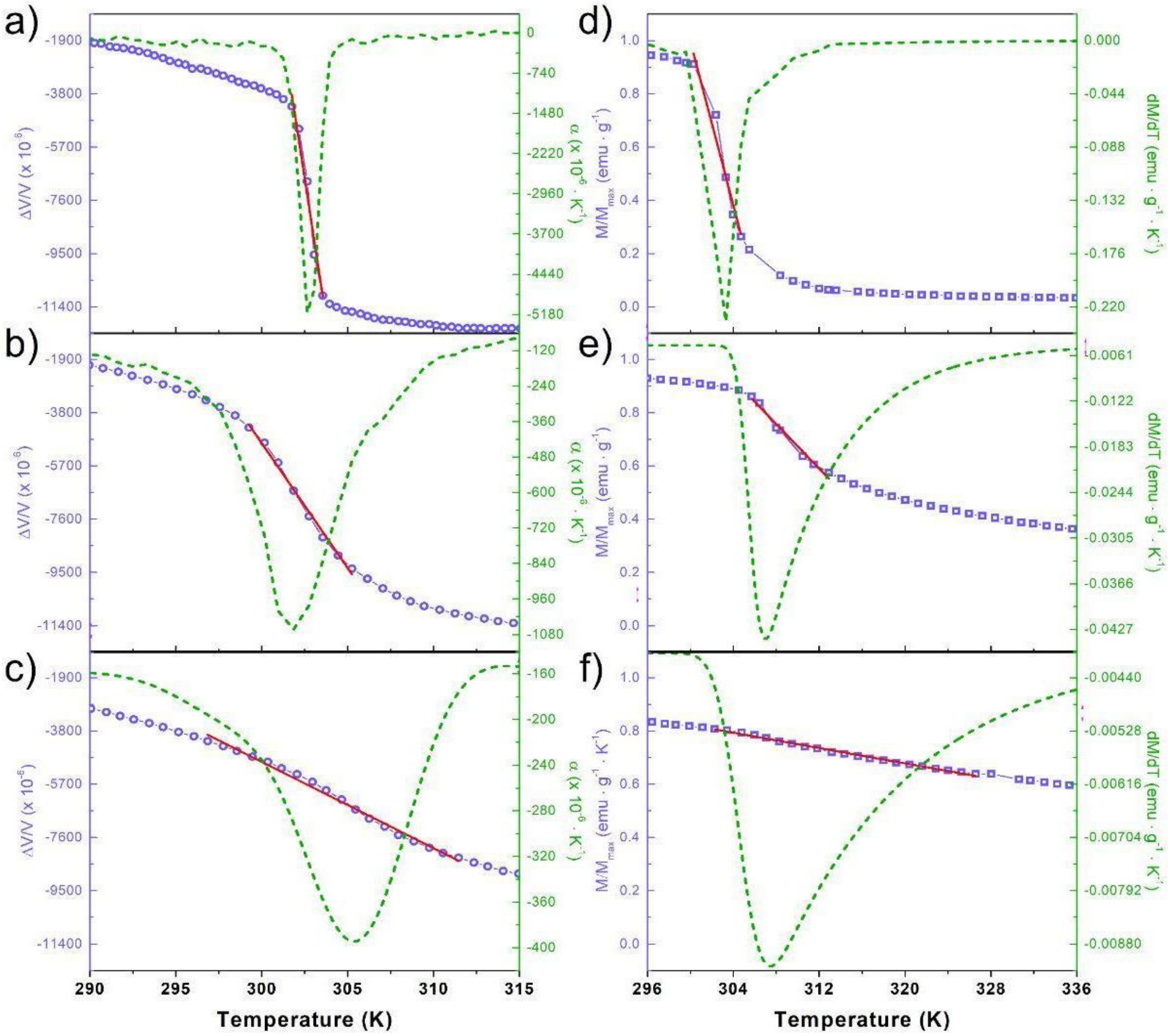

Figure S9. $\Delta V/V(T)$ (T=250 K) (blue cicles), $\alpha_V(T)(=\frac{d(\Delta V/V)}{dT})$ (green dash line) and the slope of the $\Delta V/V(T)$ analyzed at the FWHM of the $\alpha_V(T)$ curve (red line), $\underline{\alpha}_V$, for the samples a) as-prepared, b) BM45 and c) BM180. $M/M_{T=250K}$ (blue squares), $dM/dT\ (T = 250\ K)$ (green dash line) and the slope of the $M/M_{T=250K}$ analyzed at the FWHM of the $dM/dT\ (T = 250\ K)$ curve (red line), for the samples a) as-prepared, b) BM45 and c) BM180.

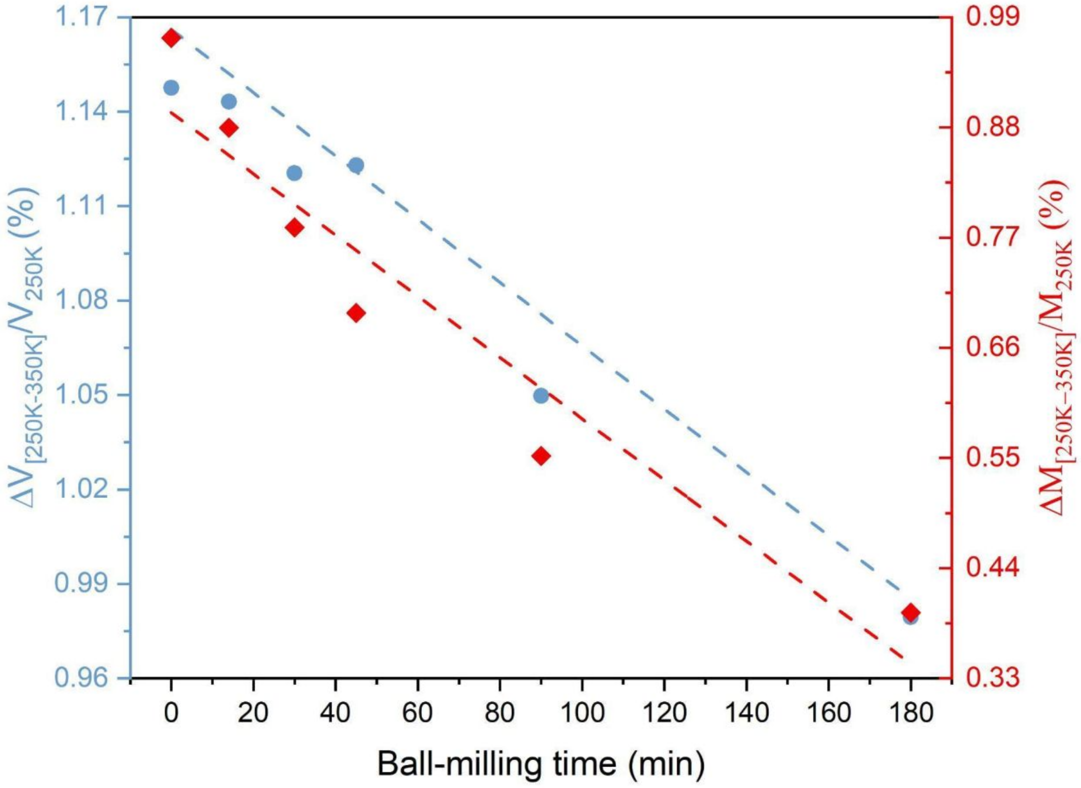

Figure S10. Relative percentual volume and magnetization change between 250K and 350K as a function of ball-milling time.

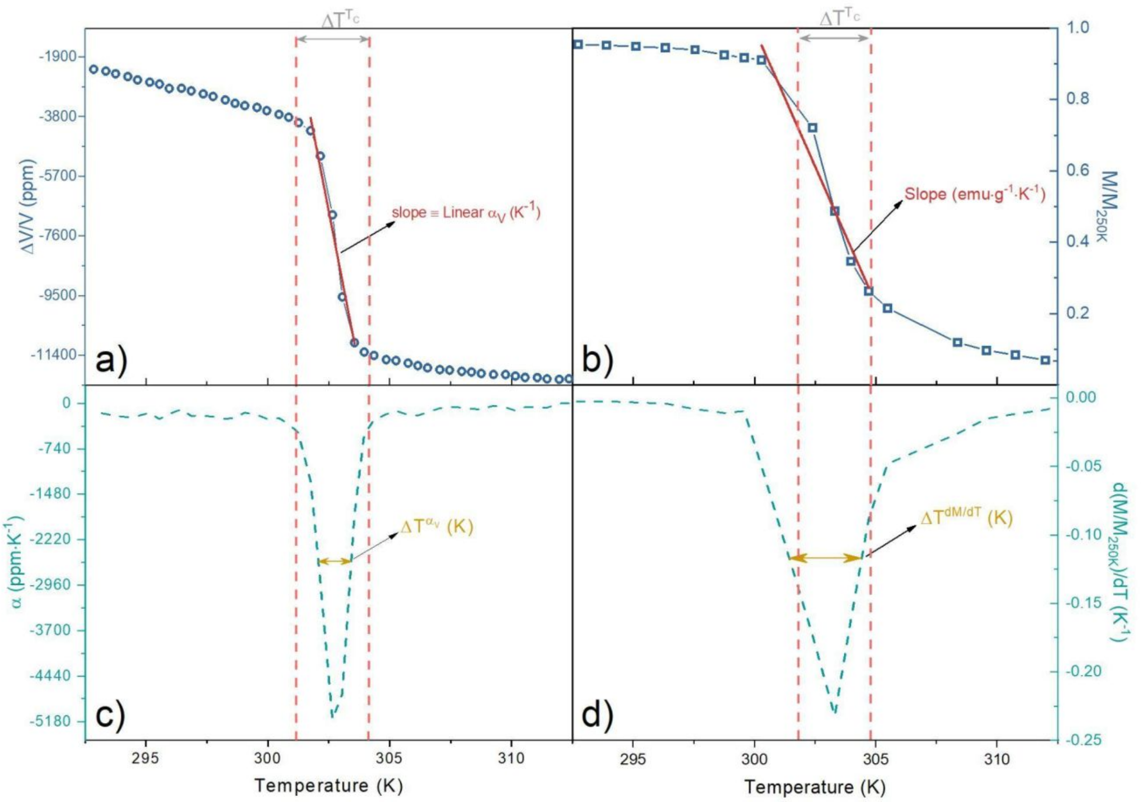

Figure S11. a) $\Delta V/V(T)$ and b) $M/M_{max}(T)$ with respective derivatives c) $\alpha(T)$, and d) $dM/dT(T)$, of the as-prepared sample. Schematic figure meant to show the temperature intervals (ΔT^{α_V}, $\Delta T^{dM/dT}$, ΔT^{T_C}) and slopes referenced throughout the paper. The red dashes represent the temperature interval ΔT^{T_C} calculated using the Williamson-Hall analysis around the samples' T_C.

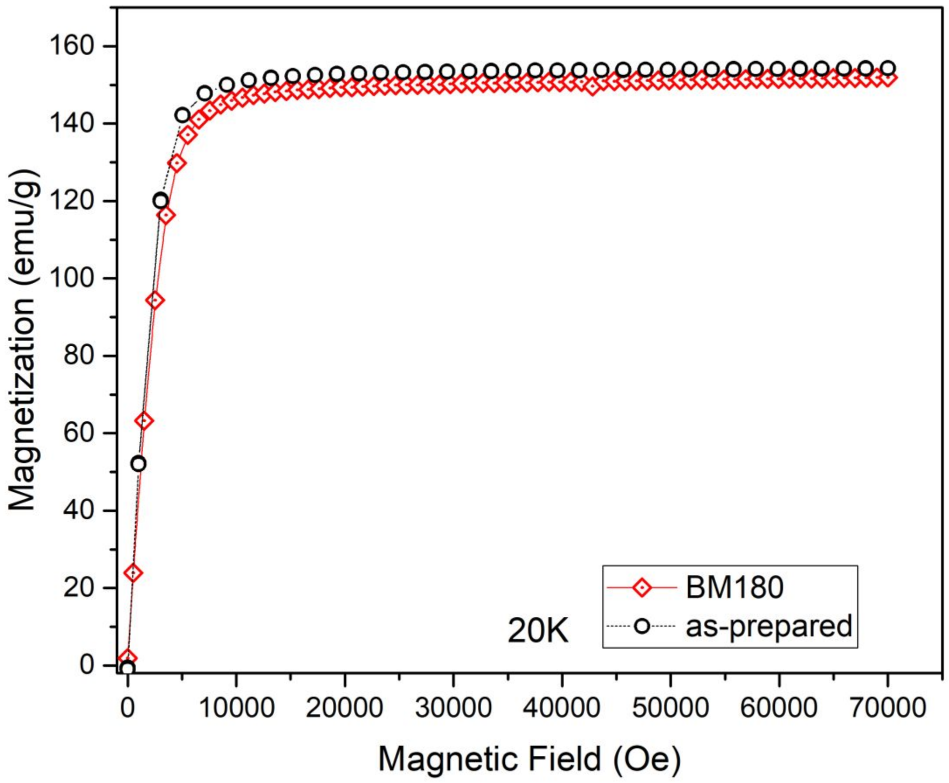

Figure S12. As-prepared and ball-milled for 180 minutes samples magnetization versus magnetic field curves measured at 20K.

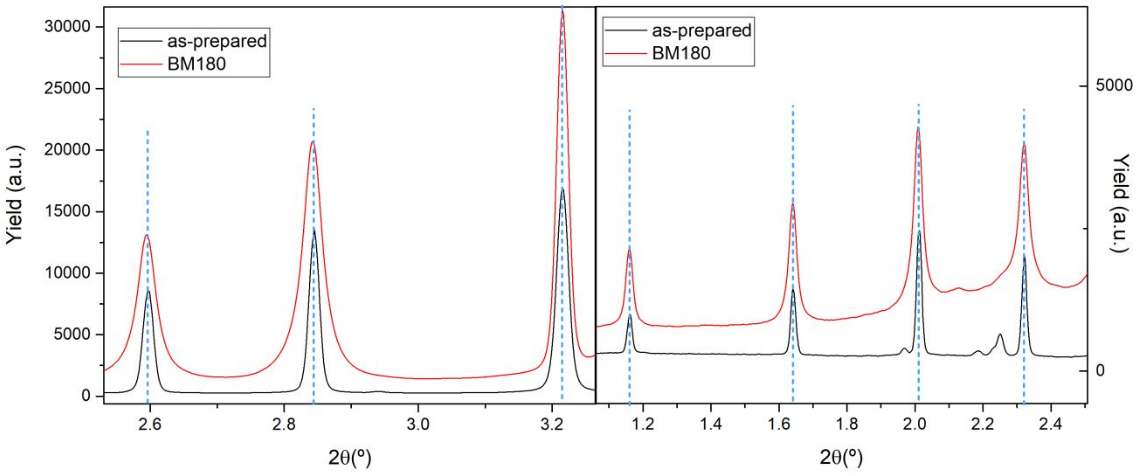

Figure S13. XRD pattern peaks of the as-prepared and ball-milled for 180 minutes samples, highlighting their identical center position.

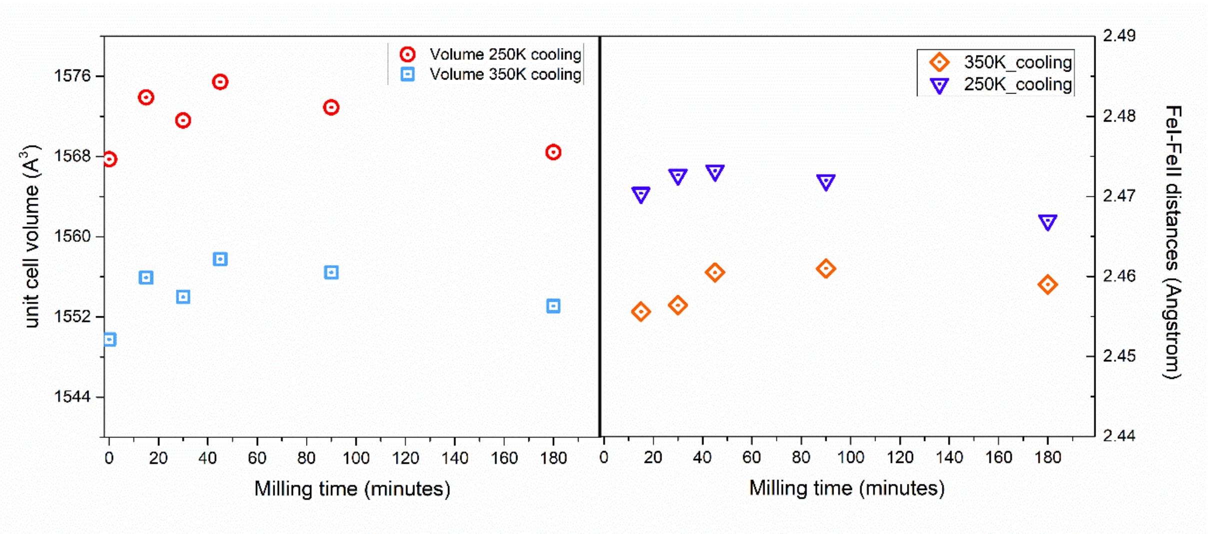

Figure S14. Unit cell volume (left-hand) and FeI-FeII interatomic distances (right-hand) obtained from the Rietveld refinements of the XRD patterns measured at low (250K) and high temperature (350K) regions as a function of ball milling time.

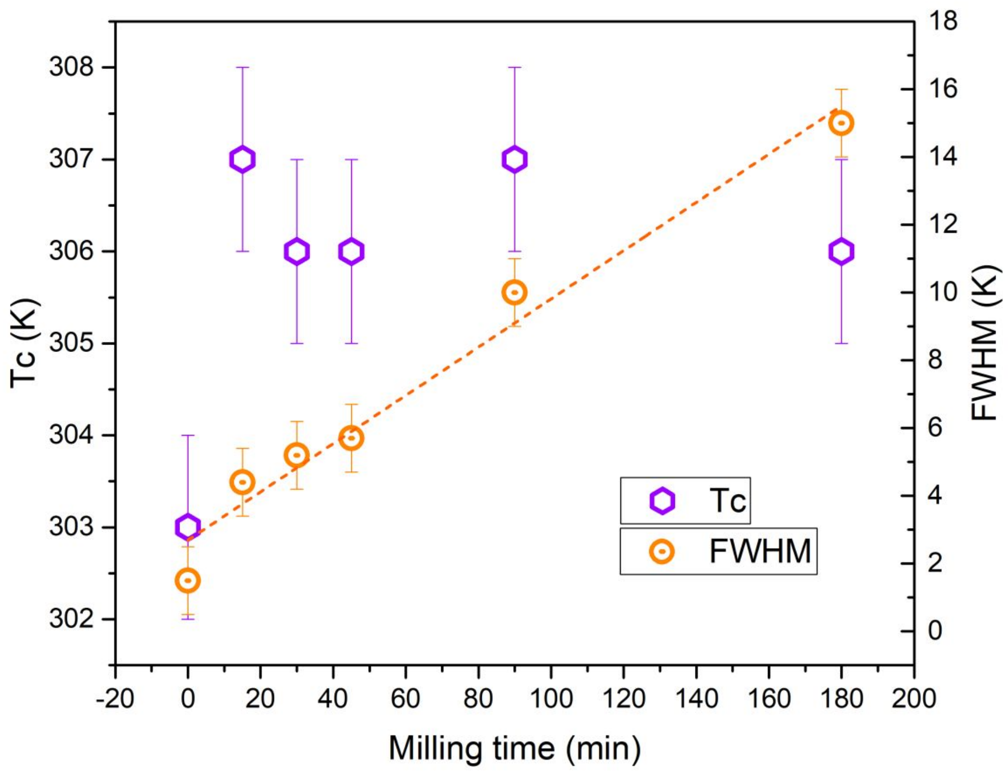

Figure S15. The magnetic transition temperature (Tc, left-axis) and the full width at half maximum of the dM/dT peak (at Tc) as a function of ball milling time.
